# Docosahexaenoic acid supplementation in gestational diabetes mellitus and neonatal metabolic health biomarkers

**DOI:** 10.3389/fnut.2023.1089131

**Published:** 2023-03-20

**Authors:** Ya-Jie Xu, Wen-Juan Wang, Qiu-Yi Zhang, Meng-Nan Yang, Lin Zhang, Hua He, Yu Dong, Fengxiu Ouyang, Ying Gao, Jun Zhang, Tao Zheng, Zhong-Cheng Luo

**Affiliations:** ^1^Department of Pediatrics, Xinhua Hospital, Ministry of Education-Shanghai Key Laboratory of Children’s Environmental Health, Early Life Health Institute, Shanghai Jiao-Tong University School of Medicine, Shanghai, China; ^2^Department of Obstetrics and Gynecology, Faculty of Medicine, Prosserman Centre for Population Health Research, Mount Sinai Hospital, Lunenfeld-Tanenbaum Research Institute, and Institute of Health Policy, Management and Evaluation, University of Toronto, Toronto, ON, Canada; ^3^Clinical Skills Center, School of Clinical and Basic Medical Sciences, Shandong First Medical University, Shandong Academy of Medical Sciences, Jinan, China; ^4^CAS Key Laboratory of Nutrition, Metabolism and Food Safety, Shanghai Institute of Nutrition and Health, Chinese Academy of Sciences, Shanghai, China; ^5^Department of Obstetrics and Gynecology, International Peace Maternity and Child Health Hospital, Shanghai Jiao-Tong University School of Medicine, Shanghai, China; ^6^Department Obstetrics and Gynecology, Xinhua Hospital, Shanghai Jiao-Tong University School of Medicine, Shanghai, China

**Keywords:** DHA (22:6*ω*–3), gestational diabetes mellitus, cord blood, prenatal intervention, adiponectin, leptin

## Abstract

**Background and objective:**

Gestational diabetes mellitus (GDM) “programs” an elevated risk of metabolic dysfunctional disorders in the offspring, and has been associated with elevated leptin and decreased adiponectin levels in cord blood. We sought to assess whether docosahexaenoic acid (DHA) supplementation in GDM affects neonatal metabolic health biomarkers especially leptin and adiponectin.

**Methods:**

In a randomized controlled trial, singleton pregnant women with *de novo* diagnosis of GDM at 24–28  weeks of gestation were randomized to dietary supplementation of 500 mg DHA per day (intervention, *n* = 30) until delivery or standard care (control, *n* = 38). The primary outcomes were cord blood leptin and total adiponectin concentrations. Secondary outcomes included high-molecular-weight (HMW) adiponectin and insulin-like growth factor-1 (IGF-1) concentrations in cord blood, maternal glycemic control post-intervention and birth weight (*z* score). In parallel, 38 euglycemic pregnant women were recruited for comparisons of cord blood biomarkers.

**Results:**

There were no significant differences in cord serum leptin, total and HMW adiponectin and IGF-1 concentrations between DHA supplementation and control groups (all *p* > 0.05). Maternal fasting and 2-h postprandial blood glucose levels at 12–16 weeks post-intervention were similar between the two groups. The newborns in the DHA group had higher birth weight z scores (*p* = 0.02). Cord blood total and HMW adiponectin concentrations were significantly lower in GDM vs. euglycemic pregnancies.

**Conclusion:**

Docosahexaenoic acid supplementation at 500  mg/day in GDM women did not affect neonatal metabolic biomarkers including leptin, adiponectin and IGF-1. The results are reassuring in light of the absence of influence on neonatal adipokines (leptin and adiponectin), and potential benefits to fetal growth and development.

**Clinical Trial Registration:**

Clinicaltrials.gov, NCT03569501.

## Introduction

Gestational diabetes mellitus (GDM) is a common pregnancy complication characterized by *de novo* hyperglycemia in the second half of pregnancy (1). About one of seven pregnancies are complicated by GDM (2). The offspring of GDM women are at elevated risks of metabolic dysfunctional disorders including obesity, type 2 diabetes, and cardiovascular diseases in later life (3–5). The mechanisms underlying such fetal “programming” remain unclear. There is lack of studies on whether any interventions may counter the adverse programming effects.

Adipose tissue dysfunction may be a mechanism in adverse developmental programming. In the human fetus, adipose tissue maturates in the second trimester, and accumulates in the third trimester of pregnancy (6). The newborns of pregnancies with maternal overnutrition are characterized by excessive fat accretion (7). The fat depots *in utero* are positively correlated with fat content in children (8),that may be related to childhood obesity. Leptin and adiponectin are important adipose tissue-secreted hormones (adipokines) in regulating energy balance and insulin sensitivity (9, 10). GDM has been associated with impaired insulin sensitivity (11), increased leptin (12, 13) and decreased adiponectin concentrations (14, 15) in newborns. It is unknown whether any prenatal interventions may affect neonatal leptin and adiponectin levels in GDM.

Docosahexaenoic acid (DHA) is a *n*–3 long chain polyunsaturated fatty acid (LCPUFA). Average dietary intakes of DHA in pregnant women nowadays are far below the dietary recommendations (16). DHA may reduce insulin resistance and triglycerides in obese children and adolescents (17) since DHA may have anti-inflammatory and insulin sensitizing properties (18, 19). Animal models have shown that DHA supplementation may decrease leptin and increase adiponectin production (20, 21). The newborns of GDM mothers tend to have low DHA levels in cord blood (22), raising the possibility of a beneficial impact of DHA supplementation on fetal metabolic health. We conducted a randomized trial to test the hypothesis that DHA supplementation in GDM may affect neonatal metabolic health biomarkers especially leptin and adiponectin.

## Materials and methods

### Study design and population

This was a single-center, open-label, randomized controlled trial. The study was approved by the Research Ethics Committee of Xinhua Hospital, Shanghai Jiao-Tong University School of Medicine, China. The trial was registered at clinicaltrials.gov (NCT03569501), and adhered to the principles of the Helsinki Declaration. Written informed consent was obtained from all participants.

DHA trial participants (*n* = 68) were enrolled in Xinhua Hospital between August 2017 and March 2019. GDM was diagnosed by a 2-h 75 g oral glucose tolerance test (OGTT) at 24–28 weeks of gestation: if the glucose values met at least one of the following criteria: fasting ≥ 5.1 mmol/l, 1-h ≥ 10.0 mmol/l, and 2-h ≥ 8.5 mmol/l, according to the International Association of the Diabetes and Pregnancy Study Groups (IADPSG) criteria (23). Women with a *de novo* diagnosis of GDM were eligible to participate if they met all the following inclusion criteria: Han Chinese, age 20–45 years, natural conception of a singleton fetus, currently not a user of fish oil or DHA self-supplementation. Exclusion criteria were maternal current severe illnesses or life-threatening conditions (e.g., cancer, renal failure, HIV, active hepatitis, tuberculosis), illicit drug users, chronic hypertension, pre-gestational diabetes, any known congenital malformation or genetic defects in the fetus.

### Randomization and intervention

Randomized assignments were based on computer-generated random numbers provided by a statistician not involved in patient screening and follow-up assessments. Participants were randomized into the intervention (*n* = 30) or control (*n* = 38) group. Participants in the intervention group were allocated six DHA capsules per day for 16 weeks, and more DHA capsules would be provided at follow-up visits whenever required until delivery. Participants in the intervention group were instructed to take six DHA capsules per day until delivery. Each capsule contains 83.2 mg DHA. Thus, the daily intake was roughly 500 mg DHA.

Subjects in both the intervention and control groups received routine standard care including regular guidance on diet and exercise in glycemic control, and glucose-lowering medication (insulin) if necessary, per standard clinical care protocol. Participants were instructed not to intake fish oil or other unplanned DHA supplements to avoid interference. There was no self-reported trial incompliance.

### Data and specimen collection

Data were collected at baseline (enrollment) and each follow-up visit (4 weeks, 8 weeks and 12–16 weeks post-intervention and at delivery) in all DHA trial participants. Maternal fasting and 2-h postprandial blood glucose (FBG and 2hPBG) concentrations were measured by a glucometer during follow-up visits in pregnancy. Maternal health and pregnancy outcomes were obtained from hospital medical records. Adverse events (unexpected symptoms or illnesses) would be recorded. Skinfold thickness at three sites (abdomen, subscapular and triceps) in the newborns was measured by a trained research staff using a Harpenden skinfold caliper. Fasting blood samples were collected at enrollment, and umbilical vein cord blood samples (1 tube without any coagulant for serum, 1 EDTA tube for plasma and erythrocytes) were collected at delivery. All collected blood samples were kept on ice, stored temporarily in a 4°C refrigerator, and centrifuged within 2 h. The separated serum and EDTA plasma samples were stored in multiple aliquots at −80°C until assays.

For comparisons of cord blood biomarkers in GDM vs. euglycemic pregnancies, we also recruited women with a euglycemic pregnancy at delivery (*n* = 38). Cord blood samples were collected, processed and stored as per protocol for participants in the DHA trial.

### Biochemical assays

Cord serum leptin was measured by an enzyme-linked immunosorbent assay (ELISA) kit from Invitrogen (Carlsbad, CA, United States). Cord serum total and high molecular weight (HMW) adiponectin were measured by an ELISA kit from ALPCO (Salem, NH, United States). Cord serum insulin-like growth factor-1 (IGF-1) was measured by an ELISA kit from Crystal Chem (Elk Grove Village, IL 60007 USA). The limits of detection were 3.5 pg./ml for leptin, 0.034 ng/ml for HMW or total adiponectin, and 2 ng/ml for IGF-1, respectively. The intra-assay and inter-assay coefficients of variation (CV) were in the range of 0.7–1.4 and < 10.1% for leptin, 0.7–4.5% and 8.9–13.6% for total and HMW adiponectin, and 5.4 and 14.7% for IGF-1, respectively.

Docosahexaenoic acid in cord blood erythrocytes was measured by Gas Chromatograph with Flame Ionization Detector (GC/FID). Briefly, lipids from 200 μl of cord blood erythrocytes were extracted in n-hexane/isopropanol with henicosanoic acid (C21:0) as the internal standard and butylated hydroxytoluene as an antioxidant. Fatty acids methyl esters (FAMEs) were obtained by incubation with methanol and sulfuric acid at 80°C for 2.5 h. After methyl esterification, FAMEs were extracted with n-hexane and remelt in isooctane. FAMEs were analyzed using an Agilent-6,890 Series with Gas Chromatograph fitted with flame ionization and a Supelco SP-2560 capillary column (100-m × 0·25-mm internal diameter; film thickness 0.20 μm; Agilent Technologies Inc.). The carrier gas was helium and the split-splitless injector was used with a split:splitless ratio of 10:1. The injection temperature was 280°C and detection temperature was 300°C. The starting temperature of the column was 90°C. After 1 min, the temperature was programmed from 90 to 170°C at a rate of 10°C/min and then from 170 to 175°C at a rate of 5°C/min. Then, the temperature was continuously increased 1°C/min up to 210°C and finally 5°C/min up to 240°C. The flow rate of gases was 0.7 ml/min. Fatty acids were identified by comparing peaks to authentic FA methyl ester standards (569 B, NuChek, Elysian, MN, United States). Twenty-one fatty acids in cord blood erythrocytes were identified as C16:0, C16:1n9, C16:1n7, C17:0, C18:0, C18:1n9, C18:1n7, C18:2n6, C20:0, C20:2n6, C22:0, C20:3n6, C20:4n6, C23:0, C24:0, C20:5n3, C24:1n9, C22:4n6, C22:5n6, C22:5n3, and C22:6n3. We used the internal control to calculate the quantity of DHA according to the following formulas. Weight of DHA (μg) = peak area of DHA/peak area of internal standard (C21:0) * weight of internal standard (C21:0). DHA (%) = weight (μg) of DHA / weight (μg) of the total fatty acids in the sample. The weight of the total fatty acids is the sum of the identified 21 fatty acids. The weight of internal standard (C21:0) is known as 76.4 μg. DHA content was expressed as weight percentage of the total 21 fatty acids, with an inter-assay CV of 4.1%.

### Outcomes

The primary outcomes were cord serum leptin and total adiponectin concentrations. The secondary outcomes included cord serum high-molecular-weight (HMW) adiponectin, insulin-like growth factor-1 (IGF-1) concentrations, maternal glycemic control (FBG and 2hPBG), and birth outcomes including birth weight, ponderal index (birth weight/birth length^3^) and the sum of abdomen, subscapular and triceps skinfold thickness. Birth weight *z* scores were calculated based on sex- and gestational age-specific Chinese fetal growth standards (24).

### Statistical analysis

The primary comparison was by intention-to-treatment analysis. Mean ± standard deviation (SD) or median and interquartile ranges were presented for continuous variables. Frequency and percentage were presented for categorical variables. *t*-Test was used to compare the differences in continuous variables between two groups. Chi-square test was used in the comparisons of categorical variables. Biomarkers with skewed crude data distributions were log-transformed in all comparisons. Pearson partial correlation coefficients with cord blood biomarkers were calculated adjusting for gestational age at delivery. All data management and analysis were conducted using SAS, version 9.4 (SAS Institute, Cary, NC, United States). The study had a power of 80.6% to detect a 0.7 SD or greater difference in cord blood leptin or adiponectin between the intervention and control groups at two-side type I error probability of 5%, with the study sample sizes (30 in DHA and 38 in control group).

## Results

### Participants and characteristics

Of the 68 participants in the trial, 30 subjects were randomized to the DHA supplementation and 38 to the control arm. Baseline characteristics of trial participants are presented in Table 1. The average age was 31.4 ± 4.6 years. Most characteristics are similar between DHA and control groups. Most (94.1%) participants received dietary intervention only for glycemic control; only 4 participants received insulin therapy (two in each arm). Participants in the DHA supplementation arm were more likely to have a caesarean section delivery (76.7 vs. 52.6%, *p* = 0.041).

**Table 1:** Characteristics of study participants in a randomized trial of docosahexaenoic acid (DHA) supplementation (500 mg/d from 24 to 28 weeks of gestation to delivery) in pregnant women with gestational diabetes mellitus (GDM).

	All (*n* = 68)	DHA (*n* = 30)	Control (*n* = 38)	*p* ^*^
Age (years)	31.4 ± 4.6	31.0 ± 4.3	31.7 ± 4.9	0.53
≥ 35	18 (26.5)	12 (31.6)	6 (20.0)	0.28
University education	38 (55.9)	16 (53.3)	22 (57.9)	0.71
Smoking	0 (0.0)	0 (0.0)	0 (0.0)	1.00
Family history of hypertension	14 (20.6)	5 (16.7)	9 (23.7)	0.48
Family history of diabetes	2 (2.9)	0 (0)	2 (5.3)	0.50
Primiparous	35 (51.5)	14 (46.7)	21 (55.3)	0.48
Gestational age at enrollment (weeks)	24.0 ± 2.3	24.1 ± 1.9	23.9 ± 2.5	0.74
Weight (kg)				
Pre-pregnancy	61.4 ± 10.6	61.9 ± 12.2	61.0 ± 9.3	0.73
At 1st trimester visit	64.3 ± 11.4	65.6 ± 12.4	63.4 ± 10.6	0.44
At delivery	73.0 ± 11.6	74.4 ± 14.2	71.9 ± 9.1	0.40
Weight gain^†^	11.6 ± 5.8	12.6 ± 6.3	10.9 ± 5.3	0.25
Height (cm)	161.8 ± 5.6	162.6 ± 5.9	161.2 ± 5.4	0.32
BMI (kg/m^2^)				
Pre-pregnancy	23.4 ± 3.5	23.3 ± 3.9	23.4 ± 3.1	0.89
At 1st trimester visit	24.5 ± 3.7	24.7 ± 3.9	24.3 ± 3.5	0.69
At delivery	27.8 ± 3.7	28.0 ± 4.4	27.6 ± 3.0	0.67
GDM treatment				0.81
Dietary intervention only	64 (94.1)	28 (93.3)	36 (94.7)	
Insulin	4 (5.9)	2 (6.7)	2 (5.3)	
Mode of delivery				**0.04**
Caesarean section	43 (63.2)	23 (76.7)	20 (52.6)	
Vaginal	25 (36.8)	7 (23.3)	18 (47.4)	

* *p* Values for comparisons between the two groups.

† Weight at delivery minus pre-pregnancy weight.

Data represented are mean ± SD or *n* (%).

BMI, body mass index; DHA, docosahexaenoic acid.

p Values in bold: *p* < 0.05.

Maternal blood concentrations of glucose, insulin and cholesterols at the first prenatal visits, GDM diagnosis in the 75 g OGTT (oral glucose tolerance test) at 24–28 weeks of gestation or the last prenatal visit are presented in Table 2. There were all similar, except for serum total triglyceride concentrations in the first prenatal visit that tended to be higher (2.1 ± 0.8 vs. 1.7 ± 0.6, *p* = 0.047) in DHA vs. control groups.

**Table 2:** Circulating concentrations of glucose, insulin and cholesterols in women with GDM in DHA supplementation vs. control groups.

	DHA (*n* = 30)	Control (*n* = 38)	*p* ^*^
First prenatal visit			
Gestational age (weeks)	14.3 ± 4.1	13.2 ± 1.9	0.16
Fasting blood glucose (mmol/L)	5.2 ± 0.7	5.0 ± 0.4	0.26
Fasting insulin (pmol/L)	49.1 (33.7–79.9)	51.7 (30.8–70.4)	0.44^†^
Total triglyceride (mmol/L)	2.1 ± 0.8	1.7 ± 0.6	**0.047**
Total cholesterol (mmol/L)	4.8 ± 0.8	4.6 ± 0.9	0.34
HDL cholesterol (mmol/L)	1.8 ± 0.4	1.9 ± 0.5	0.84
LDL cholesterol (mmol/L)	2.4 ± 0.6	2.2 ± 0.6	0.44
Creatinine (umol/L)	42.0 ± 5.1	42.9 ± 6.4	0.56
75 g OGTT at 24–28 weeks of gestation			
Fasting blood glucose (mmol/L)	5.2 ± 0.6	5.0 ± 0.4	0.26
1-h blood glucose (mmol/L)	9.6 ± 1.3	9.6 ± 1.7	0.87
2-h blood glucose (mmol/L)	7.6 ± 1.7	7.3 ± 1.7	0.57
Fasting insulin (pmol/L)	66.6 (43.2–80.9)	58.8 (34.5–88.3)	0.73^†^
2-h insulin (pmol/L)	356.3 (190.2–504.8)	339.6 (275.6–513.3)	0.78^†^
HbA1c (%)	5.4 ± 0.7	5.2 ± 0.4	0.11
Last visit in late gestation			
Gestational age (weeks)	36.0 ± 7.3	33.7 ± 4.6	0.16
Fasting blood glucose (mmol/L)	4.8 ± 0.6	4.8 ± 0.6	0.94
HbA1c (%)	5.9 ± 1.2	5.5 ± 0.5	0.18
Total triglyceride (mmol/L)	3.3 ± 1.3	3.3 ± 1.0	0.99
Total cholesterol (mmol/L)	5.9 ± 1.1	5.9 ± 1.0	0.96
Creatinine (μmol/L)	45.7 ± 7.4	44.4 ± 6.4	0.48

* *p* Values in *t*-tests for comparisons between the two groups.

† *p* Values for comparisons between the two groups in log-transformed data for insulin.

Data represented are mean ± SD or median (IQR).

DHA, docosahexaenoic acid; GDM, gestational diabetes mellitus; HbA1c, glycated hemoglobin; HDL, high-density lipoprotein; LDL, low-density lipoprotein.

*p* Values in bold: *p* < 0.05.

### Cord blood biomarkers

There were no significant differences in cord serum leptin, total and HMW adiponectin, and IGF-1 concentrations between the DHA supplementation vs. control groups (Table 3). There were no significant differences in cord serum leptin, total and HMW adiponectin, and IGF-1 concentrations comparing cesarean section vs. vaginal deliveries (all *p* > 0.20; Supplementary Table S1), or comparing male vs. female newborns (all *p* > 0.20; Supplementary Table S2).

**Table 3:** Cord blood DHA in erythrocytes and serum biomarkers in women with GDM in DHA supplementation vs. control groups.

	DHA (*n* = 30)	Control (*n* = 38)	*p* ^1^	*p* ^2^
DHA in erythrocytes (%)^*^	6.62 ± 1.05	6.45 ± 0.96	0.51	0.29
	6.50 (6.05–7.08)	6.45(5.86–7.13)		
Serum				
Leptin (ng/ml)	9.0 ± 6.4	8.9 ± 5.3	0.66	0.46
	7.5 (3.8–14.5)	8.0 (4.2–13.4)		
HMW adiponectin (μg/ml)	7.0 ± 2.9	8.2 ± 4.1	0.37	0.50
	6.3 (4.8–9.1)	7.5 (5.1–11.2)		
Total adiponectin (μg/ml)	14.5 ± 4.0	15.3 ± 5.2	0.69	0.85
	14.4 (11.3–16.9)	14.9 (12.2–17.8)		
IGF-1 (ng/ml)	28.7 ± 10.9	29.8 ± 10.2	0.60	0.50
	26.2 (21.5–33.7)	30.0 (22.7–35.6)		

* DHA content was expressed as weight percentage of total fatty acids in erythrocytes.

1 Crude *p* values from *t*-tests; log-transformed data were used for serum biomarkers.

2 *p* Values adjusted for mode of delivery from generalized linear models; other co-variables did not affect the comparisons.

Data presented are mean ± SD and median (inter quartile range).

DHA, docosahexaenoic acid; GDM, gestational diabetes mellitus; HMW, high-molecular-weight; IGF-1, insulin-like growth factor-1.

Adjusting for gestational age at birth, strong positive correlations were observed between cord serum IGF-1 and birth weight *z*-score (*r* = 0.49, *p* = 0.006), ponderal index (*r* = 0.52, *p* = 0.004) or the sum of abdomen, subscapular and triceps skinfold thickness (*r* = 0.51, *p* = 0.005; Supplementary Table S3). Cord serum total adiponectin was positively correlated with ponderal index (*r* = 0.41, *p* = 0.025). There are no correlations between cord blood DHA and leptin, adiponectin, or IGF-1.

### Birth outcomes

Birth outcomes in DHA supplementation vs. control groups are presented in Table 4. There were marginal non-significant increase in head circumference (mean: 34.6 vs. 34.0 cm, *p* = 0.08) and the sum of abdomen, subscapular and triceps skinfold thickness (15.7 vs. 14.4 mm, *p* = 0.10), and significant increases in birth weight (3,494 vs. 3,259 g, *p* = 0.04), birth weight *z* score (0.5 vs. –0.1, *p* = 0.02) and ponderal index (27.9 vs. 26.9 kg/m^3^, *p* = 0.03). There were no differences in the duration of gestation, preterm birth (< 37 weeks) and post-term birth (> 40 weeks) rates.

**Table 4:** Birth outcomes in DHA supplementation versus control groups.

	DHA (*n* = 30)	Control (*n* = 38)	*p* ^*^
Female, sex	16 (53.3)	19 (50.0)	0.79
Gestational age at birth(weeks)	39.0 ± 0.9	39.1 ± 1.2	0.62
Preterm birth (< 37 weeks)	1 (3.3)	1 (2.6)	1.00
Post-term birth (> 40 weeks)	1 (3.3)	2 (5.3)	0.70
Birth weight (g)	3,494 ± 489	3,259 ± 420	**0.04**
*Z* score^†^	0.5 ± 1.2	-0.1 ± 1.0	**0.02**
Birth length (cm)	49.9 ± 1.6	49.4 ± 1.7	0.22
Ponderal index (kg/m^3^)	27.9 ± 2.3	26.9 ± 1.5	**0.03**
Head circumference (cm)	34.6 ± 1.5	34.0 ± 1.1	0.08
Skinfold thickness (mm)			
Abdomen	4.4 ± 1.0	4.1 ± 0.8	0.14
Subscapular	5.1 ± 1.2	4.7 ± 0.9	0.10
Triceps	6.1 ± 1.5	5.6 ± 1.0	0.08
Total^‡^	15.7 ± 3.7	14.4 ± 2.6	0.10

† Based on sex- and gestational age-specific Chinese fetal growth standards.

‡ The sum of abdomen, triceps and subscapular skinfold thicknesses.

* *p* Values in *t*-test for comparisons between the two groups.

Data represented are mean ± SD or *n* (%).

*p* Values in bold: *p* < 0.05.

**Figure 1:**
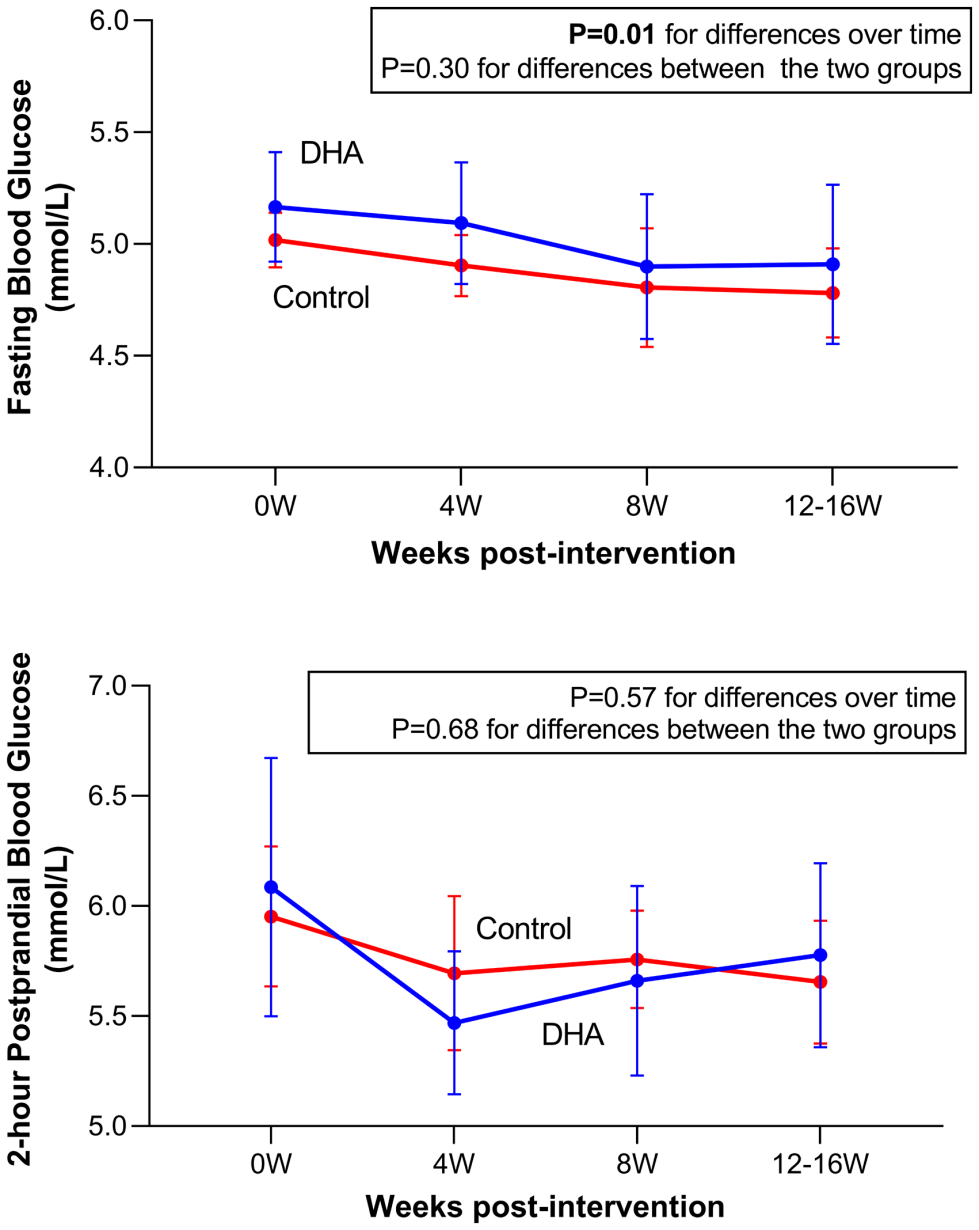
Fasting and 2-h postprandial blood glucose concentrations (mean ± 95% CI) at 0, 4, 8, and 12–16 weeks post-intervention in women with gestational diabetes mellitus (GDM) in docosahexaenoic acid (DHA) supplementation and control groups.

Maternal glucose data during follow-ups are presented in Figure 1 and Supplementary Table S4. DHA supplementation did not affect the changes in maternal FBG and 2hPBG concentrations from baseline (enrollment) to 12–16 weeks post-intervention (delivery). There was a trend toward lower FBG concentrations from baseline to 12–16 weeks post-intervention in both DHA and control groups.

### Compliance and safety

All subjects reported compliance to the intervention protocol. DHA content (%) in cord blood erythrocytes was non-significantly higher in the DHA vs. control groups (mean ± SD: 6.62 ± 1.05 vs. 6.45 ± 0.96, adjusted *p* = 0.29). No adverse events were reported.

### Gestational diabetes mellitus vs. euglycemic pregnancies

Comparing GDM vs. euglycemic pregnancies, average pre-pregnancy BMI was higher (mean: 23.4 vs. 21.6 kg/m^2^), gestational age at delivery was lower (mean: 39.0 vs. 39.7 weeks), while other maternal and neonatal characteristics were similar (Supplementary Table S5). Cord serum total and HMW adiponectin concentrations were lower in GDM vs. euglycemic pregnancies, while leptin and IGF-I concentrations were similar (Supplementary Table S6).

## Discussion

### Main findings

The study is the first randomized controlled trial to explore the effect of DHA supplementation on neonatal leptin and adiponectin levels in GDM pregnancies. We observed that DHA supplementation at 500 mg/day from 24 to 28 weeks of gestation to delivery in GDM pregnancies did not affect cord blood concentrations of leptin, HMW and total adiponectin and IGF-1 in newborns. However, it was associated with enhanced fetal growth.

### Cord blood metabolic health biomarkers

To our knowledge, there have been only two randomized controlled trials evaluating whether n-3 LCPUFAs supplementation alters neonatal leptin and/or adiponectin levels, and both were conducted in healthy pregnant women (25, 26). Batirel et al. (25) studied pregnant women with n-3 LCPUFAs daily supplementation [eicosapentaenoic acid (EPA) 504 mg + DHA 378 mg; *n* = 13] and controls (without supplementation, *n* = 18) from 22 to 24 weeks of gestation to delivery, and reported no effect on cord blood leptin. England et al. (26) studied pregnant women with DHA-rich fish oil supplementation (containing 90 mg DHA, *n* = 37) or soy oil (placebo controls, *n* = 41) from 12 to 20 weeks gestation until delivery, and reported no alterations in cord blood leptin and adiponectin concentrations. The findings of these two studies are consistent with our results, although our trial used a higher dose of DHA supplementation. It is encouraging that DHA supplementation at higher dose (500 mg/d) did not affect cord blood leptin and adiponectin concentrations in our study, considering the potential benefits to fetal growth and development.

Surprisingly, cesarean section deliveries were more frequent comparing DHA supplementation vs. control groups. In a large randomized controlled trial of DHA supplementation during pregnancy (*n* = 2,399), DHA supplementation was associated with an increased risk of post-term induction or cesarean section delivery (RR = 1.28) that might be attributable to prolonged gestation (27). However, we observed no differences in the duration of gestation and post-term birth rate between DHA supplementation and control groups (Table 4). More studies are warranted to clarify whether DHA supplementation may increase the risk of cesarean section delivery. However, cesarean section did not affect cord serum leptin, adiponectin and IGF-I concentrations (Supplementary Table S1), and therefore would not have affected our comparisons.

### Birth weight

We observed higher birth weight (*z* score) in the DHA supplementation (at 500 mg/d) group. This finding is consistent with the results in a trial of DHA supplementation at 600 mg/d in the second half of pregnancy (28), and in a DHA-rich fish oil supplementation trial (29, 30). It has been suggested that DHA may alter the balance of prostaglandins to prolong gestation and thus increase birth weight (31, 32). However, we did not detect longer duration of gestation in the DHA supplementation group, suggesting a direct positive impact on fetal growth. The lack of difference in cord serum IGF-I concentration suggests that the positive impact of DHA on fetal growth might be mediated by factors other than IGF-I.

Similar to the previous trial of 600 mg/day DHA supplementation in GDM women (33), fatty acids in erythrocytes revealed only non-significant increases in neonatal DHA in the supplementation group in our trial. It is possible that DHA supplementation at 500–600 mg/day from GDM diagnosis (at 24–28 weeks of gestation) to delivery may be insufficient in resulting in substantial improvements in neonatal DHA status according to DHA content in cord blood erythrocytes. Alternatively, there might be un-reported non-compliances that could have obscured the differences.

The positive impact of DHA on fetal growth may be through promoting angiogenesis in the placenta (34) and increasing the prostacyclin to thromboxane ratio enhancing placental–fetal blood flow. DHA is a structural fatty acid playing an important role in the optimization of brain development. Prenatal DHA supplementation in pregnant women may improve offspring’s cognitive function and attention (35, 36). In this study, we observed that the newborns of women with GDM had a smaller head circumference compared to those of euglycemic pregnant women (*p* = 0.004), raising a concern on the impact of GDM on neurodevelopment. In addition, average head circumference was marginally increased (*p* = 0.08) in DHA supplementation vs. control groups in our study, suggesting a potential beneficial impact of DHA on neurodevelopment.

### Limitations

The main study limitation is the relatively small sample size. The study was powered to detect relatively large differences (≥ 0.7 SD), but not powered to detect small differences. The study was open-labeled, and self-reported compliance was excellent, but we could not rule out the possibility of un-reported non-compliances. Lastly, we did not have data on long-term postnatal follow up outcomes.

### Conclusion

Our trial suggests that DHA supplementation at 500 mg/day in the 3rd trimester of pregnancy in GDM women does not affect cord blood metabolic health biomarkers including leptin, adiponectin and IGF-1. The results are reassuring in light of the absence of influence on neonatal adipokines (leptin and adiponectin), and potential benefits to fetal growth and development.

## Data availability statement

The datasets presented in this article are not readily available because Access to the deidentified participant research data must be approved by the research ethics board on a case-by-case basis. Requests to access the datasets should be directed to Z-CL (zc_luo@yahoo.com); TZ (zhengtao@xinhuamed.com.cn).

## Ethics statement

The studies involving human participants were reviewed and approved by Xinhua Hospital, Shanghai Jiao-Tong University School of Medicine, Shanghai, China. The patients/participants provided their written informed consent to participate in this study.

## Author contributions

Z-CL, TZ, JZ, YG, FO, and LZ conceived the study. Y-JX, W-JW, Q-YZ, M-NY, LZ, HH, YD, TZ, and Z-CL contributed to the acquisition of research data. Y-JX, W-JW, Q-YZ conducted the literature review, data analysis and drafted the article. Z-CL is the guarantor of this work, has full access to all the data in the study and takes responsibility for the integrity of the data and the accuracy of the data analysis. All authors contributed in revising the article critically for important intellectual content, and approved the final version for publication.

## Funding

This work was supported by research grants from the Ministry of Science and Technology of China (2019YFA0802501), the Shanghai Municipal Science and Technology Commission (21410713500), and the Shanghai Municipal Health Commission (2020CXJQ01).

## Conflict of interest

The authors declare that the research was conducted in the absence of any commercial or financial relationships that could be construed as a potential conflict of interest.

## Publisher’s note

All claims expressed in this article are solely those of the authors and do not necessarily represent those of their affiliated organizations, or those of the publisher, the editors and the reviewers. Any product that may be evaluated in this article, or claim that may be made by its manufacturer, is not guaranteed or endorsed by the publisher.

## Abbreviations

GDM: gestational diabetes mellitus
DHA: docosahexaenoic acid
LCPUFA: long-chain polyunsaturated fatty acids
IGF-1: insulin-like growth factor-1
HMW: high-molecular-weight
FBG: fasting blood glucose
2hPBG: 2-h postprandial blood glucose
