# Supplementary material for: Docosahexaenoic acid supplementation in gestational diabetes mellitus and neonatal metabolic health biomarkers
Source: Front Nutr. 2023 Mar 20;10:1089131. doi: 10.3389/fnut.2023.1089131 (PMC10069675; doi:10.3389/fnut.2023.1089131)
Supplement: Supplementary file 1 [file Data_Sheet_1.docx]

**Appendix - Supplementary Tables S1-S6**

**TABLE S1.** Cord serum leptin, total and HMW adiponectin, and IGF-1 concentrations comparing cesarean section vs. vaginal deliveries

|  | **Cesarean section (n=43)** | **Vaginal**  **(n=25)** | **P*** |
| --- | --- | --- | --- |
| Leptin (ng/ml) | 9.7±6.0 | 7.8±5.3 | 0.26 |
| HMW adiponectin (μg/ml) | 7.3±3.3 | 8.4±4.2 | 0.31 |
| Total adiponectin (μg/ml) | 14.5±4.6 | 15.6±4.9 | 0.36 |
| IGF-1 (ng/ml) | 30.1±11.4 | 27.8±8.5 | 0.54 |

Data presented are mean±SD.

IGF-1, insulin-like growth factor-1; HMW, high-molecular-weight.

*P values comparing the two groups from independent samples t-tests in log-transformed data.

**TABLE S2.** Cord serum leptin, total and HMW adiponectin, and IGF-1 concentrations comparing male vs. female newborns

|  | **Male (n=33)** | **Female (n=35)** | **P*** |
| --- | --- | --- | --- |
| Leptin (ng/ml) | 8.6±5.3 | 9.3±6.2 | 0.81 |
| HMW adiponectin (μg/ml) | 15.7±5.2 | 14.2±4.2 | 0.42 |
| Total adiponectin (μg/ml) | 8.1±4.0 | 7.3±3.3 | 0.29 |
| IGF-1 (ng/ml) | 28.9±10.4 | 29.7±10.7 | 0.82 |

Data presented are mean±SD.

IGF-1, insulin-like growth factor-1; HMW, high-molecular-weight.

*P values comparing the two groups from independent samples t-tests in log-transformed data.

**TABLE S3.** Correlations of DHA content in cord blood erythrocytes with serum biomarkers, birth weight, ponderal index and skinfold thickness

|  | Cord blood DHA* | | Birth weight (z) | | Ponderal index | | Skinfold thickness^‡^ | |
| --- | --- | --- | --- | --- | --- | --- | --- | --- |
|  | r | P | r | P | r | P | r | P |
| Cord blood DHA |  |  | 0.08 | 0.68 | 0.04 | 0.85 | 0.13 | 0.52 |
| Cord serum |  |  |  |  |  |  |  |  |
| Leptin | 0.05 | 0.81 | 0.32 | 0.09 | 0.35 | 0.06 | 0.30 | 0.11 |
| HMW adiponectin | -0.04 | 0.86 | 0.07 | 0.71 | 0.23 | 0.23 | 0.02 | 0.93 |
| Total adiponectin | 0.02 | 0.91 | 0.29 | 0.11 | 0.41 | **0.03** | 0.30 | 0.11 |
| IGF-1 | -0.13 | 0.52 | 0.49 | **0.006** | 0.52 | **0.004** | 0.51 | **0.005** |

Data presented are correlation coefficients adjusting for gestational age at birth.

IGF-1, insulin-like growth factor-1; HMW, high-molecular-weight.

*DHA was expressed as weight percentage of total fatty acids in cord blood erythrocytes.

**^‡^**The sum of abdomen, triceps and subscapular skinfold thicknesses.

P values in **bold**: P< 0.05.

**TABLE S4.** Comparisons of maternal blood glucose concentrations from enrollment to 12 weeks post-intervention in DHA (n=30) versus control (n=38) groups

|  | Visit* | DHA | Control | P value for the effect of | | |
| --- | --- | --- | --- | --- | --- | --- |
|  |  |  |  | DHA | Visit | Interaction |
| Fasting blood glucose | 1 | 5.2±0.6 | 5.0±0.4 | 0.30 | **0.01** | 0.89 |
| (mmol/L) | 2 | 5.1±0.7 | 4.9±0.3 |  |  |  |
|  | 3 | 4.9±0.7 | 4.8±0.5 |  |  |  |
|  | 4 | 4.9±0.9 | 4.8±0.6 |  |  |  |
| 2-h postprandial blood glucose | 1 | 6.1±1.5 | 6.0±0.9 | 0.68 | 0.57 | 0.63 |
| (mmol/L) | 2 | 5.5±0.8 | 5.7±0.9 |  |  |  |
|  | 3 | 5.7±0.9 | 5.8±0.6 |  |  |  |
|  | 4 | 5.8±0.8 | 5.7±0.6 |  |  |  |

Data presented are Mean±SD.

* Visit 1/2/3/4：at 0/4/8/12 weeks post-intervention.

P values were from two-way analysis of variance; P values in **bold**: p<0.05.

**TABLE S5.** Maternal and neonatal characteristics in GDM and euglycemic pregnancies.

|  | Euglycemic (n= 38) | GDM (n= 68) | P* |
| --- | --- | --- | --- |
| Mothers |  |  |  |
| Age (years) | 31.5±4.5 | 31.4±4.6 | 0.98 |
| ≥35 | 9 (23.7) | 18 (26.5) | 0.75 |
| BMI (kg/m^2^) |  |  |  |
| Pre-pregnancy | 21.6±3.2 | 23.4±3.5 | **0.01** |
| At delivery | 27.7±2.8 | 27.8±3.7 | 0.85 |
| Education, university | 24 (63.2) | 38 (55.9) | 0.47 |
| Family history of hypertension | 10 (26.3) | 14 (20.6) | 0.50 |
| Family history of diabetes | 2 (5.23) | 2 (2.9) | 0.62 |
| Primiparous | 21 (55.3) | 35 (51.5) | 0.71 |
| Gestational age at recruitment (weeks) | 23.7±2.5 | 24.0±2.3 | 0.59 |
| Newborns |  |  |  |
| Caesarean section delivery | 28 (73.7) | 43 (63.2) | 0.27 |
| Female sex | 18 (47.4) | 35 (51.5) | 0.69 |
| Gestational age (weeks) | 39.7±0.8 | 39.0±1.1 | **0.002** |
| Birth weight (g) | 3398.2±332.7 | 3362.6±463.6 | 0.65 |
| z-score^†^ | 0.0±0.8 | 0.1±1.1 | 0.55 |
| Birth length (cm) | 49.8±1.5 | 49.6±1.7 | 0.66 |
| Ponderal index (kg/m^3^) | 27.5±1.9 | 27.3±1.9 | 0.69 |
| Head circumference (cm) | 34.8±1.1 | 34.3±1.3 | 0.06 |
| Skinfold thickness (mm) |  |  |  |
| Abdomen | 4.1±0.6 | 4.2±0.9 | 0.52 |
| Subscapular | 4.8±0.7 | 4.9±1.0 | 0.50 |
| Triceps | 5.6±0.8 | 5.8±1.3 | 0.27 |
| Total^‡^ | 14.5±2.1 | 15.0±3.2 | 0.40 |
| NICU admission | 1 (2.7) | 6 (8.8) | 0.42 |
| Preterm birth (< 37 weeks) | 0 (0.0) | 2 (2.9) | 0.54 |
| Cord blood DHA (%) ^a^ | 6.71±0.86 | 6.52±1.00 | 0.34 |

Data represented as Mean±SD or n (%). BMI, body mass index.

^a^ DHA content as weight percentage of total fatty acids in erythrocytes.

**†** Based on sex- and gestational age-specific Chinese fetal growth standards.

‡ The sum of abdomen, triceps, and subscapular skinfold thicknesses.

* P values for comparisons between the two groups. P values in **bold**: p<0.05.

**TABLE S6.** Cord serum biomarkers in GDM vs. euglycemic pregnancies

|  | **Euglycemic**  **(n= 38)** | **GDM**  **(n=68)** | **P^1^** | **P^2^** |
| --- | --- | --- | --- | --- |
| Leptin (ng/ml) | 8.9±4.7 | 9.0±5.8 | 0.57 | 0.73 |
|  | 7.8 (5.2-12.1) | 7.6 (4.2-13.5) |  |  |
| HMW adiponectin (μg/ml) | 10.2±3.5 | 7.7±3.6 | **0.001** | **0.01** |
|  | 10.3 (7.9-12.5) | 7.0 (4.8-9.6) |  |  |
| Total adiponectin (μg/ml) | 19.0±5.2 | 14.9±4.7 | **<0.001** | **0.001** |
|  | 18.9 (15.5-22.0) | 14.7 (11.4-17.8) |  |  |
| IGF-1 (ng/ml) | 28.1±10.4 | 29.3±10.5 | 0.51 | 0.87 |
|  | 27.8 (21.2-33.5) | 27.3 (21.7-35.2) |  |  |

Data presented are mean±SD and median (IQR).

IGF-1, insulin-like growth factor-1; HMW, high-molecular-weight.

All P values were from comparisons between the two groups in log-transformed biomarkers data.

^1^P values from independent samples t-tests in log-transformed data.

^2^P values adjusted for pre-pregnancy BMI, gestational age at birth from generalized linear models; other co-variables did not affect the comparisons.

P values in **bold**: p<0.05.
